# Rapid, ultrasensitive and highly specific diagnosis of *Mycoplasma pneumoniae* by a CRISPR-based detection platform

**DOI:** 10.3389/fcimb.2023.1147142

**Published:** 2023-07-27

**Authors:** Juan Zhou, Fei Xiao, Jin Fu, Nan Jia, Xiaolan Huang, Chunrong Sun, Zheng Xu, Yu Zhang, Dong Qu, Yi Wang

**Affiliations:** ^1^ Experimental Research Center, Capital Institute of Pediatrics, Beijing, China; ^2^ Department of Critical Medicine, Children’s Hospital Affiliated Capital Institute of Pediatrics, Beijing, China

**Keywords:** *Mycoplasma pneumoniae*, MP infection, recombinase polymerase amplification, CRISPR, Cas12b

## Abstract

*Mycoplasma pneumoniae* (MP) is an important causative agent of morbidity and mortality among all age groups, especially among patients of extreme ages. Improved and readily available tests for accurate, sensitive and rapid diagnosis of MP infection is sorely needed. Here, we developed a CRISPR-Cas12b-based detection platform on the basis of recombinase polymerase amplification (RPA) for rapid, simple, and accurate diagnosis of MP infection, named MP-RPA-CRISPR. The RPA was employed for amplifying the community-acquired respiratory distress syndrome (CARDS) toxin gene of MP strains at the optimal reaction temperature 37°C. The resulting amplicons were decoded by the CRISPR-Cas12b-based detection platform, which was interpreted by real-time PCR system and by naked eye under blue light. The MP-RPA-CRISPR can detected down to 5 fg of genomic DNA templates of MP strains and accurately distinguish MP strains from non-MP strains without any cross-reactivity. A total of 96 bronchoalveolar lavage fluid (BALF)samples collected from patients suspected of respiratory infection were used to evaluate the clinical performance of the MP-RPA-CRISPR assay. As a result, our assay accurately diagnosed 45 MP-infected samples and 51 non-MP infected sample, and the results obtained from MP-RPA-CRISPR were consistent with microfluidic chip technology. In conclusion, our MP-RPA-CRISPR assay is a simple, rapid, portable and highly sensitive method to diagnose MP infection, which can be used as a promising tool in a variety of settings including clinical, field, and resource-limited aeras.

## Introduction

*Mycoplasma pneumoniae* (MP) is one of the most common causative agents of respiratory infections in humans, accounting for 4% to 8% of community-acquired bacterial pneumonia (CABP). During epidemics, the incidence of CABP due to MP infection could climb up to 20%-40% (Shankar et al., 2007; Loens et al., 2010; Jacobs et al., 2015; Bajantri et al., 2018; Krafft and Christy, 2020). Clinical manifestations of MP infection were commonly presented as fever, cough, myalgias and/or gastrointestinal symptoms, which were mild and non-specific. However, more severe presentations, including bronchospasm, pneumonia, acute respiratory distress syndrome (ARDS), myocarditis, meningitis and so on, were also have been reported (Smith et al., 1980; Narita, 2010; Mishra et al., 2017). Particularly, it was reported that about 12% of the hospitalized children with MP infection were admitted to the ICU (Kutty et al., 2019). However, due to its asymptomatic or non-specific characteristics and lack of optimal diagnostic tests with good sensitivity and specificity, the prevalence of MP infection was largely underestimated and it was difficult to assess the true impact of MP on public health. Thus, diagnostic methods with superior sensitivity and specificity were highly encouraged to help diagnose and treat MP infection.

Current laboratorial diagnosis methods for MP infection included culture, serological test and various nucleic acid amplification-based assays (including conventional PCR and real-time PCR) (Waites et al., 2017). Although considered as the traditional “gold standard” for diagnosis of MP infection (China National Health Commission, 2023), culture of MP was time-consuming and not readily available (Daxboeck et al., 2003), serological tests and PCR-based assays thus were the main tools for the diagnosis of MP infection in clinical practices. Of note, it was reported that combination of serological tests and PCR-based assays in diagnosis of MP infection could largely improve the reliability and accuracy of MP detection (Loens and Ieven, 2016). However, in addition to not suitable for the patients of extreme ages (infants and the elderly), the serological tests for the diagnosis of MP infections are somewhat nonspecific and may cross-react with other pathogens (such as Epstein-Barr virus, cytomegalovirus or *Klebsiella*) (Bajantri et al., 2018). Although PCR-based methods were considered highly sensitive and specific, they were unable to be performed without sophisticated systems or thermocycling procedures, which were commonly equipped in well-established laboratories. Therefore, more rapid, flexible and sensitive diagnostic platforms need to be further developed to meet the need for field-deployable diagnostic tests of MP infection.

The clustered regularly interspaced short palindromic repeats and their associated proteins (CRISPR-Cas) system, which typically is considered as an adaptive immune defense mechanism against invasive foreign elements (DNA or RNA sequences) in many archaea and bacteria, has been increasingly introduced to the genome editing and nucleic acid diagnosis fields owing to its simple to develop, highly sensitive, and independence of dedicated instruments (Li et al., 2019; Liu et al., 2022). The CRISPR-Cas system achieves nucleic acid detection by leveraging the “collateral activity’’ of the Cas effectors, such as the single-strand DNA (ssDNA) cleavage activity of Cas12a and Cas12b effector (Chen et al., 2018; Li et al., 2019) or the single-strand RNA (ssRNA) cleavage activity of Cas13 effector (Gootenberg et al., 2017). Recently, the CRISPR-Cas platform coupling with several isothermal amplification techniques, including recombinase polymerase amplification (RPA) (Piepenburg et al., 2006; Gong et al., 2022), loop-mediated isothermal amplification (LAMP) (Notomi et al., 2000) and multiple cross displacement amplification (MCDA) (Wang et al., 2015; Zhou et al., 2023), have been developed for rapid and sensitive detection of various pathogens (Chen et al., 2018; Li et al., 2019; Chen et al., 2021; Gong et al., 2022).

RPA is one of the booming isothermal nucleic acid amplification technique that was developed by Armes’s et al. in 2006 (Piepenburg et al., 2006). Typically, the RPA technique could achieve exponential amplification of target DNA with high sensitivity and specificity after incubated at 37 to 42°C for less than 30 min. Moreover, the RPA reagents are commonly freeze-dried, the RPA kit could be stored at room temperature instead of a cold-chain. All of these allow the RPA assay available for POC (point-of-care) or “on-site” detection of pathogen at a mobile suitcase labora18tory.

Here, we thus incorporated RPA with CRISPR-Cas12b detection platform to develop a new MP testing, termed MP-RPA-CRISPR. In this report, we illustrated the basic principle of the MP-RPA-CRISPR assay, and evaluated its analytical sensitivity, and specificity as well as validated its clinical feasibility using the clinical samples collected from suspected MP-infected patients.

## Materials and methods

### Primer and gRNA design

The RPA primers, which targeted the community-acquired respiratory distress syndrome (CARDS) toxin gene (LR214945.1) (10), were designed by Primer Premier 5.0 software. Hairpin, dimer, false priming and cross dimer of the primers were also identified by Primer Premier 5.0 software. The primer specificity was assessed by the BLAST program of the National Center for Biotechnology Information (NCBI) database. The target product was a segment of 224 bp. Moreover, a gRNA based on the CARDS gene of MP strains was designed on the basis of the CRISPR-Cas12b detection mechanism. Location and sequence of the primers and gRNA were shown in **Figure S1**. All of the oligomers were synthesized and purified by TianYi-HuiYuan Biotech. Co., Ltd. (Beijing, China) with an HPLC purification grade. gRNA was synthesized by GeneScript Biotech. Co, Ltd. (Nanjingjing, China) at HPLC purification grade.

### Bacterial strains and DNA preparation

A total of 26 bacterial strains, including MP reference strain MP129 and 25 non-MP strains of other respiratory bacterial pathogens (**Table S1**), were used in this study. Cells of strain MP129 was inoculated in mycoplasma agar (Thermo Fisher Oxoid, Basingstoke, UK) and incubated at 37°C for more than 10 days. Growth was monitored by observing their size and shape on the plates. Identification was performed by microfluidic chip technology (CapitalBio Technology, Chengdu, China) which was commercially available. Genomic DNA of all the strains were extracted and purified using the EasyPure^®^ Genomic DNA Kit (Beijing TransGen Biotech Co., Ltd, Beijing, China) according to the manufacture’s instruction. All the extracted DNA samples were stored at -20°C before use.

### Recombinase polymerase amplification reaction

The RPA reaction was performed using a commercial recombinase polymerase-based amplification kit (Msunflowers Biotech Co., Ltd, Beijing, China). The reaction mixture was of 50 μl, including 41.5 μl of A buffer, 2 μl each of the primers, 2 μl of template and 2.5 μl of B buffer. After incubated at 37°C for 30 min, the resultant products were monitored by agarose gel electrophoresis (AGE, 2%) method to verify the availability of the selected primers. Genomic DNA of *M. pneumoniae* and *Klebsiella pneumoniae* (KP) were used as positive and negative controls, and distilled water (DW) as blank control. In addition, the reaction temperature of RPA was further optimized by evaluating the amplification efficiency conducted within the range of 37 to 42°C with 1°C intervals.

### CRISPR-Cas12b detection

The RPA products was detected by the CRISPR-Cas12b-based detection system. The CRISPR-Cas12b-based detection system was a volume of 25 μl mixture, including 12.5 μl 2× Cas12b buffer, 8 μl AapCas12b-gRNA complex, 1 μl ssDNA fluorescence probe (FAM-TTATTATTAT-BHQ1, 100 μM), 2 μl RPA product, and 1.5 μl nuclease-free water. The AapCas12b-gRNA complex was prepared by mixing 1.5 μl AapCas12b (10 μM, HUIDEXIN) and 2 μl gRNA (10 μM) up in 2 × Cas12b buffer and incubated at 37°C for 10 min, followed by immediately used or stored at 4°C no more than 12 h. The result was monitored by the real-time PCR system or by naked eyes under blue light using the Imager System. Results of target cleavage by the CRISPR-Cas12b-gRNA complex were verified with AGE method as well. In order to identify the accurate complex for trans-cleavage reaction, different combinations of the three key components, i.e., CRISPR-Cas12b, gRNA, target DNA, were subjected to examination. The result was displayed in the format of fluorescence intensity.

What’s more, the reaction condition of CRISPR-Cas12b detection was further optimized by exploring the optimal volume of CRISPR-Cas12b-gRNA complex and the suitable time for trans-cleavage reaction. The optimal volume of CRISPR-Cas12b-gRNA complex for CRISPR-Cas12b detection of RPA products was determined by examining and comparing the produced fluorescent signal after addition of 3, 4, 5, 6 and 8 μl of CRISPR-Cas12b-gRNA complex, respectively. The suitable time for trans-cleavage reaction was confirmed by comparing the produced fluorescent signal within a reaction time of 5 min, 10 min, 15 min and 20 min, respectively.

### Sensitivity and specificity evaluation of the MP-RPA-CRISPR assay

To assess the sensitivity of the MP-RPA-CRISPR assay, the genomic DNA of MP strain M129 was 10-fold serially diluted from 5 ng to 0.5 fg to conduct the MP-RPA-CRISPR assay, with DW as blank control. The specificity of the assay was evaluated by testing the genomic DNA of MP strain MP129 and 25 non-MP strains (**Table S1**). Each test was repeated three times.

### Clinical feasibility confirmation of the MP-RPA-CRISPR assay

A total of 96 clinical samples were used to confirm the feasibility of the MP-RPA-CRISPR assay for MP detection in clinical settings. The clinical samples were bronchoalveolar lavage fluid (BALF)samples collected from patients suspected of respiratory infection based on clinical symptoms, and they have been detected by microfluidic chip technology for the causative agents diagnosis. The DNA templates of these clinical samples were extracted by nucleic acid extraction reagent purchased from CapitalBio Technology Co., Ltd (Sichuan, China). Aliquots of 5 μl of DNA templates were used to perform the MP-RPA-CRISPR assay.

### Statistical analysis

All the experimental results are shown as mean ± SEM unless otherwise stated. Student’s t-test was used to assess statistical significance when only two groups were compared. Significance was displayed as ∗, *P* < 0.05; ∗∗, *P* < 0.01; ∗∗∗, *P* < 0.001; and ∗∗∗∗, *P* < 0.0001. Statistical analyses were conducted using GraphPad Prism 8.0.

## Results

### Schematic of the MP-RPA-CRISPR assay

**Figure 1:**
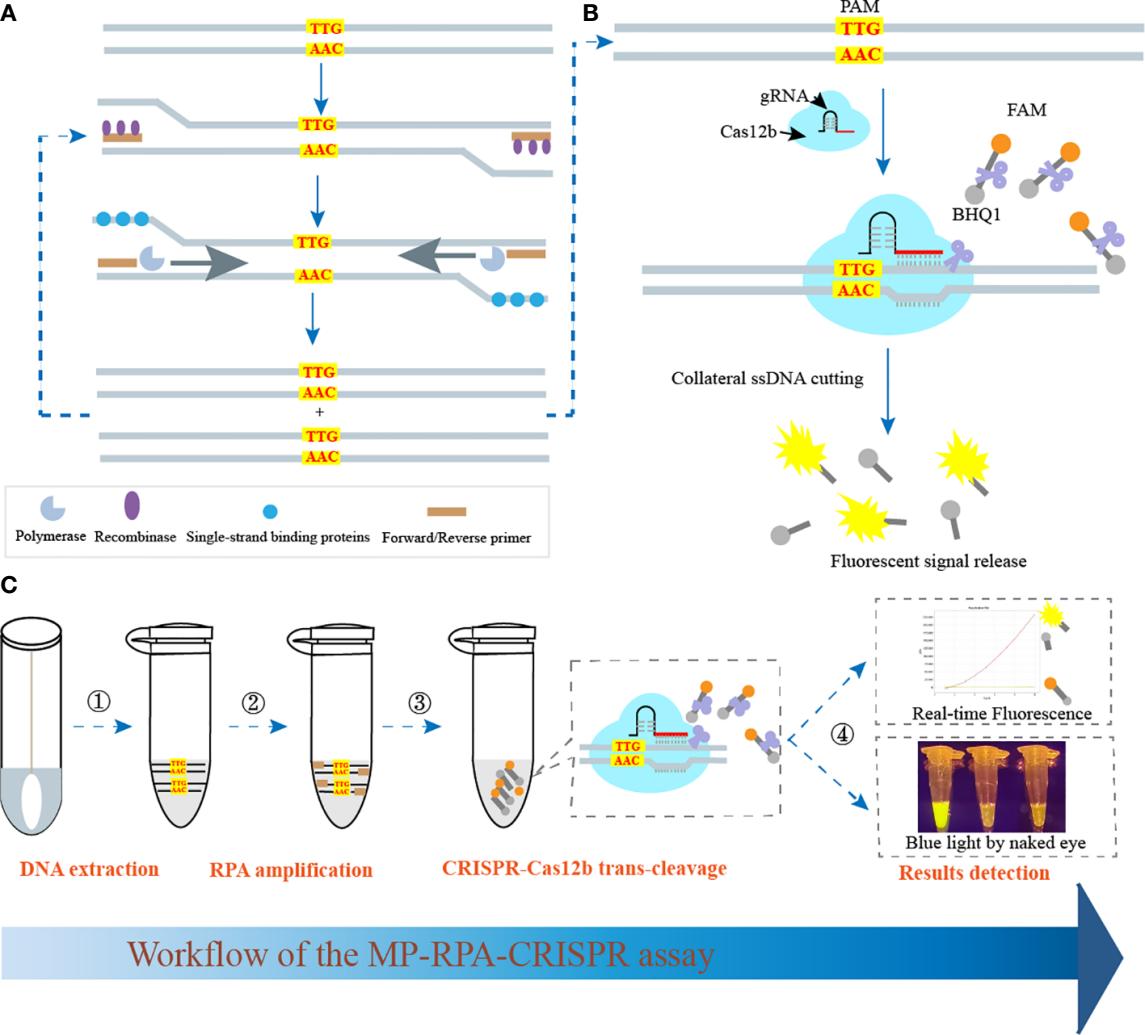
Schematic illustration of MP-RPA-CRISPR assay’s principle. **(A)** Principle of RPA assay. After RPA reaction, a plenty of amplicons containing PAM site specific for CRISPR-Cas12b (TTG) were produced. **(B)** Principle and procedure of the CRISPR-Cas12b-based trans-cleavage detection. After the pre-amplification by RPA, the PAM site within the resultant amplicons was recognized by the CRISPR-Cas12b/gRNA complex and formed a ternary complex, which was then activated and nonspecifically cut the FAM/BHQ1 labeled probe, resulting to the release of fluorescent signals. **(C)** Workflow of the MP-RPA-CRISPR assay. The whole assay included four steps, including DNA extraction, RPA reaction, CRISPR-Casa12b tans-cleavage and results reporting. The interpretation of results reporting could be performed by both real-time PCR system and by naked eye under blue light.

The schematic illustration of the MP-RPA-CRISPR assay was shown in **Figure 1**. The MP-RPA-CRISPR detection system, which combined RPA with CRISPR-Cas12b-based detection platform, was carried out for MP infection diagnosis. Briefly, the target gene containing a PAM site (TTG) specific for Cas12b was amplified by the RPA kit at 37°C, then, a plenty of products containing the PAM site were produced (**Figure 1**). The resulting products could be recognized by the corresponding Cas12b/gRNA complex (**Figure 1**). Once the PAM sites in RPA products were recognized, the trans-cleavage activity of AapCas12b was rapidly activated, followed by the nonspecific cleavage of the ssDNA probes, resulting to release the fluorescent signals (**Figure 1**). The released fluorescent signals could be monitored by a real-time PCR system or by naked eyes under blue light using the Imager System (**Figure 1**).

### Verification of the MP-RPA-CRISPR assay

**Figure 2:**
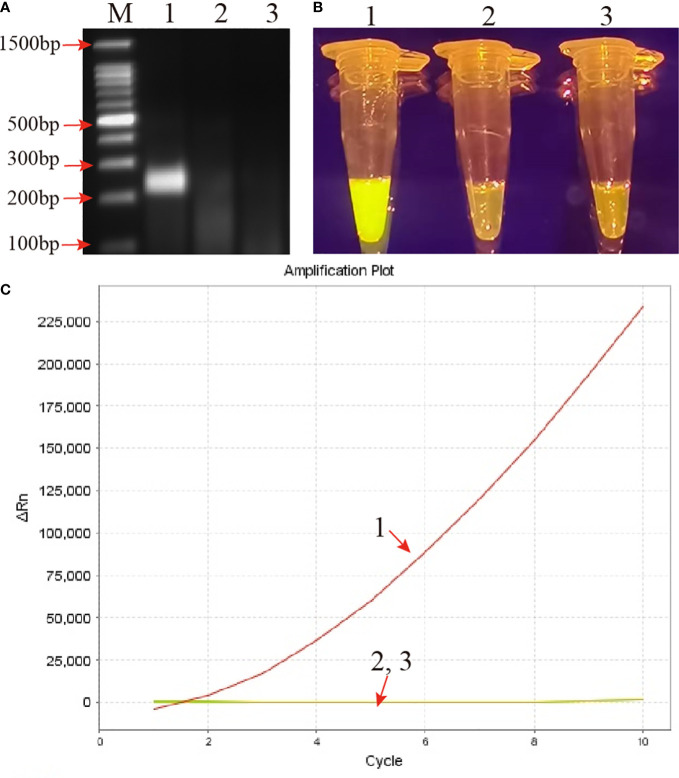
Confirmation and validation of the MP-RPA-CRISPR assay. **(A)** Confirmation of the amplification products of RPA by agarose gel electrophoresis (AGE) method. **(B)** Confirmation of the amplification products of RPA using CRISPR-Cas12b-based detection platform by naked eye under blue light. **(C)** Confirmation of the amplification products of RPA using CRISPR-Cas12b-based detection platform by real-time PCR system. Templates of 1-3 were the genomic DNA of MP, KP and DW, respectively. M represented the 100 bp DNA marker.

Genomic DNA of the MP strain M129 was selected to verify the reliability of the RPA primers. After amplified at 37°C for 30 min, the products were examined by 2% AGE and CRISPR-Cas12b detection platform simultaneously. By AGE, a visible band was observed in the mixture with MP strain M129 as template, while no band was observed in the products of KP and DW (**Figure 2**). Likewise, using the CRISPR-Cas12b detection platform, positive results were observed only in the RPA products of MP. The reaction tube exhibited bright yellow when it visualized by naked eye under blue light (**Figure 2**). In addition, apparent fluorescence signals can be observed when the CRISPR-Cas12b detection was monitored by real-time PCR system (**Figure 2**). Therefore, the primers used in this study was suitable for development of MP-RPA-CRISPR assay.

Besides, after CRISPR-Cas12b cleavage, two shorter bands (133bp and 91 bp) were obtained in the reaction mixture when visualized with AGE method (**Figure S2A**), implying that the CRISPR-Cas12b effector was capable of cutting double-strand DNA (dsDNA). In addition, by comparing the trans-cleavage results of different key component combinations (**Figure S2B**), it was obvious that the formation of complex of CRISPR-Cas12b-gRNA-target DNA played a pivotal role for target DNA detection.

### Optimal conditions for MP-RPA-CRISPR assay

In MP-RPA-CRISPR system, the optimum reaction temperature during pre-amplification stage was determined by performing RPA reaction at various temperatures, ranging from 37 to 42°C with 1°C increments. As shown in **Figure S3A**, although all temperatures showed good amplification efficiency for the MP-RPA assays, 37°C was selected for the subsequent MP-RPA-CRISPR assay according to the recommended temperature from kit manufacturer. As shown in **Figures S3B, C**, the optimal volume of CRISPR-Cas12b-gRNA complex was of 8 μl and a reaction time of 10 min were optimal for RPA products trans-cleavage. The resultant optimal reaction conditions were applied in the following detections.

### Sensitivity and specificity of the MP-RPA-CRISPR assay

**Figure 3:**
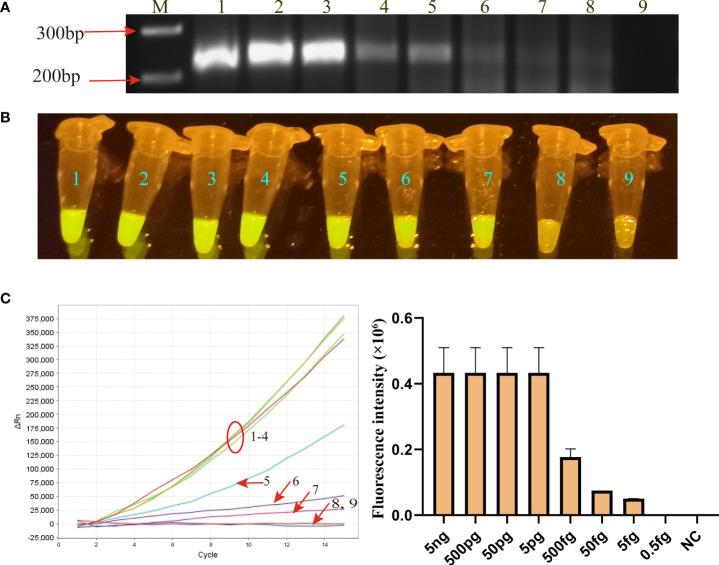
Sensitivity of MP-RPA-CRISPR assay. **(A)** Sensitivity of the MP-RPA assay detected by AGE method. **(B)** Sensitivity of the MP-RPA-CRISPR assay visualized by naked eye under blue light. **(C)**, Sensitivity of the MP-RPA-CRISPR assay recorded by the real-time PCR system. 1–9 represented the genomic DNA templates of MP strain M129 ranging from 5 ng to 0.5 fg and that of Kp. The LoD level of MP-RPA-CRISPR assay was 5 fg per reaction, and that for RPA assay visualized by AGE was 50 fg per reaction.

Using the serially diluted MP genomic DNA templates, the limit of detection (LoD) of the MP-RPA-CRISPR assay was evaluated. As shown in **Figure 3**, the LoD of MP-RPA reaction by CRISPR-Cas12b-based detection platform was lower to 5 fg per reaction (**Figure 3B** **)**, while that of directly detected by AGE method was of only 50 fg per test (**Figure 3**). Thus, the MP-RPA-CRISPR assay was able to detect as low as 5 fg of MP genomic DNA per reaction.

**Figure 4:**
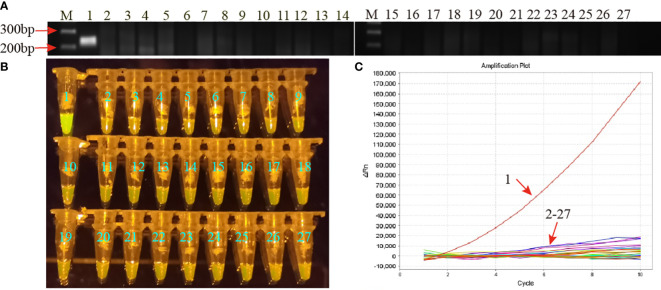
Specificity of the MP-RPA-CRISPR assay. **(A)** Specificity of the MP-RPA assay detected by AGE method. **(B)** Specificity of the MP-RPA-CRISPR assay visualized by naked eye under blue light. **(C)** Specificity of the MP-RPA-CRISPR assay recorded by the real-time PCR system. 1 represented the genomic DNA templates of MP strain M129, 2-27 represented the genomic DNA templates of 25 non-MP pathogens and DW. Only the genomic DNA of the MP strain M129 was positively detected by the MP-RPA-CRISPR assay.

Specificity of the MP-RPA-CRISPR assay was assessed using genomic DNA templates of 25 non-MP strains, including 5 strains of other members of genus *Mycoplasma* and 20 common respiratory pathogens in clinic (**Table S1**). Positive results were only observed in the mixture of MP strain M129, whereas all the non-MP strains and DW showed negative results (**Figure 4**). No cross-reactions were observed from the MP-RPA-CRISPR assay, indicating a specificity of 100% of the newly developed assay.

### Clinical feasibility of the MP-RPA-CRISPR assay

**Figure 5:**
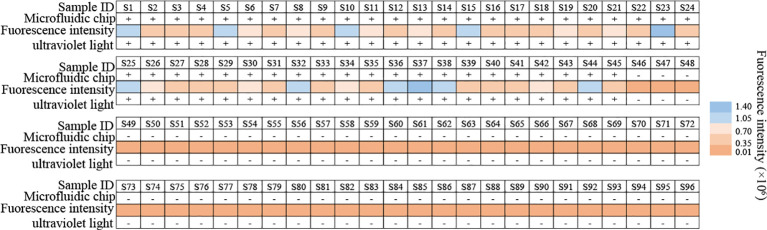
Clinical feasibility validation of the MP-RPA-CRISPR assay. A total of 96 clinical samples were used to confirm the clinical performance of the MP-RPA-CRISPR assay. All the positive samples that tested by microfluidic chip technology showed positive results when detected by the MP-RPA-CRISPR assay, and the negative ones displayed negative results as well. S1-S96 represented the 96 bronchoalveolar lavage fluid (BALF) samples collected from patients suspected of respiratory infection based on clinical symptoms.

Clinical feasibility of the MP-RPA-CRISPR assay was examined using the templates extracted from 45 BALF samples from 45 MP infected patients and 51 non-MP infected BALF samples, all of which have been detected using microfluidic chip technology for clinical diagnosis. The results of the MP-RPA-CRISPR assay for these samples were in accordance with those by microfluidic chip technology, with the 45 positive samples positive for MP and the 51 negative ones negative for MP (**Figure 5**, **Figure S4**). Thus, the analytical sensitivity and specificity of the MP-RPA-CRISPR assay were all 100%, indicting the MP-RPA-CRISPR assay was feasible for MP infection diagnosis in clinic.

## Discussion

MP infection remains an important etiology of morbidity and mortality among all age groups, especially among patients of extreme ages (1). Along with the increasing emergence of macrolides resistant MP strains in many areas of the world, controlling, treating and surveilling MP infection have become more complicated. Thus, more work is still needed to design improved and readily available tests for accurate, sensitive and rapid diagnosis of MP infection. Here, we developed a CRISPR-Cas12b-based detection platform combining the isothermal RPA technique (termed MP-RPA-CRIPSR) to achieve the quick, sensitive, specific and easy diagnosis of MP infection.

CRISPR/Cas system has exhibited great potential in nucleic acid detection filed in recent years owning to the advantages of high sensitivity, specificity, portability and simplicity. Among the several reported Cas effectors (such as Cas12a, Cas12b, and Cas13), Cas12b has sparked great interest because of the merit of having a wide range of reaction temperature for trans-cleavage (Li et al., 2019; Li et al., 2021). Due to the relatively low detection sensitivity of Cas protein detection alone, Cas12b effector was usually employed *via* combining with various isothermal amplification methods to improve the detection sensitivity (Li et al., 2019; Chen et al., 2021; Li et al., 2021). In this report, the CRISPR-Cas12b-based detection system was coupled with isothermal amplification technique RPA to improve the performance of common RPA in the diagnosis of MP infection (**Figures 1A** **)**. Particularly, the PAM site specific for Cas12b (TTN) to recognize target sequences was located within the target sequences (**Figure S1**), eliminating the additional introduction of an engineered primers. Moreover, followed the untargeted cleavage of fluorescence and quencher labeled ssDNA reporter, the CRISPR-Cas12b-based biosensing system could transfer sequence information into fluorescent signals, which could be detected by real-time PCR system or more portable fluorescence reader (such as by naked eye under blue light) (**Figure 1**), endowing this method with great potential in POC or “on-field” diagnosis. Thus, the whole detection process, including DNA extraction, PRA pre-amplification, CRISPR/Cas12 cleavage and results detection, could achieve MP diagnosis more quickly, sensitively, specifically and portably.

In this study, a remarkable advantage of the MP-RPA-CRISPR assay was its simple to operation and independence of sophisticated instruments and cold-chain. The pre-amplification of target nucleic acid sequences by RPA could be performed only using a heat container that maintained a constant temperature of 37 to 42°C. Moreover, the RPA regents were freeze-dried, thus the RPA kit can be stored at room temperature for several months instead of cold chain. In addition, the RPA products detection in this assay was implemented by the CRISPR-Cas12b-based detection platform. The MP-RPA-CRISPR assay employed a FAM and fluorescent quencher (BHQ1) labeled ssDNA reporter to interpret the RPA amplification results. Comparing the standard real-time detection for RPA assay, the results of the MP-RPA-CRISPR assay could not only be monitored by real-time PCR system, but also could be visualized by naked eyes under blue light, which was of vital importance for POC diagnostics, especially in the conditions lack of expensive instruments.

With regard to the analytical performance, as envisaged, the MP-RPA-CRISPR assay was highly sensitive in the MP detection. This assay could detect as low as 5 fg of genomic DNA templates of MP strains (equivalent to 5 *M. pneumoniae* cells), which was comparable to that based on CRISPR-Cas12a biosensing system (Li et al., 2022) but apparently superior than those detecting RPA products by AGE (50fg, **Figure 3**) or by real-time fluorescent probe (10 fg) (Jiang et al., 2022), as well as other isothermal amplification-based methods, such as the LAMP-based assay (50 fg) (Xiao et al., 2022) and the MCDA-based assay (50 fg) (Wang et al., 2019). In addition, the MP-RPA-CRISPR assay was extremely specific for MP detection with a specificity of 100%. It correctly distinguished the clinically common respiratory pathogens and other members of genus *Mycoplasma* from MP, and no cross-activity was observed. All of these indicated that the MP-RPA-CRISPR assay developed here was highly sensitive and specific for MP detection.

In terms of clinical feasibility, the MP-RPA-CRISPR assay exhibited excellent performance in diagnosis of MP infection. The MP-RPA-CRISPR assay accurately identified the 45 MP-infected samples, which was identical to the results detected by microfluidic chip technology, a commercially available method with high throughput for parallel identification of multiple pathogens related to clinical pneumonia (Huang et al., 2017). Given the concordance between the MP-RPA-CRISPR assay and the microfluidic chip technology, the MP-RPA-CRISPR assay showed high reliability for MP detection. What’s more, the 51 negative samples detected by microfluidic chip technology also displayed negative results in the MP-RPA-CRISPR assay. These results demonstrated that the MP-RPA-CRISPR assay has great potential to become promising candidates for MP detection tool in clinics and POC tests.

Due to the importance and urgency of MP infection control, several new methods for rapid, simple, sensitive and specific detection of MP infection have been established in our laboratory (Xiao et al., 2022; Jia et al., 2023), and we are committed to devising a more portable, rapid, accurate and sensitive MP detection platform. Compared with our previous developed LAMP- and MCDA-based methods (50 fg), the new RPA-based method devised here was more sensitive (5 fg) and portable to diagnose MP infection. Of course, there are several drawbacks in our study: detection of RPA results by CRISPR-Cas12b detection system needs to open the reaction tube, which may increase the opportunity of cross-contaminations. Thus, the risk of carryover contamination should be further evaluated for ensuring the reliability. Moreover, careful and quick operation in separate clean areas or even developing one-step reaction assay are also encouraged measures to reduce the risks.

In conclusion, we developed a CRISPR-Cas12b-based detection platform following the RPA reaction for rapid and accurate detection of MP and termed it as MP-RPA-CRISPR assay. The assay’s results could be detected by real-time PCR systems or visualized by naked eye under blue light. The data of analytical sensitivity, specificity, and clinical feasibility assessment demonstrated that the new protocol is capable of diagnosing MP infection simply, rapidly, portably and accurately with high sensitivity and specificity. Thus, these merits of the MP-RPA-CRISPR assay enable this assay a promising tool for MP detection in a variety of settings including clinical, field, and resource-limited aeras.

## Data availability statement

The original contributions presented in the study are included in the article/**Supplementary Material**. Further inquiries can be directed to the corresponding authors.

## Ethics statement

Ethical review and approval was not required for the study on human participants in accordance with the local legislation and institutional requirements. Written informed consent from the participants was not required to participate in this study in accordance with the national legislation and the institutional requirements.

## Author contributions

JZ performed the experiments and drafted the manuscript. FX, NJ, and XH helped analyze the data. JF, CS, ZX, and YZ provided reagents and materials. YW and DQ conceived this study, supervised clinical guidance, and revised the manuscript. All authors contributed to the article and approved the submitted version.

## References

[B1] Bajantri B. Venkatram S. Diaz-Fuentes G. (2018). Mycoplasma pneumoniae: A potentially severe infection. J. Clin. Med. Res. 10 (7), 535–544. doi: 10.14740/jocmr3421w 29904437 PMC5997415

[B2] Chen J. S. Ma E. Harrington L. B. Da Costa M. Tian X. Palefsky J. M.. (2018). CRISPR-Cas12a target binding unleashes indiscriminate single-stranded DNase activity. Science 360 (6387), 436–439. doi: 10.1126/science.aar6245 29449511 PMC6628903

[B3] Chen X. Tan Y. Wang S. Wu X. Liu R. Yang X.. (2021). A CRISPR-cas12b-based platform for ultrasensitive, rapid, and highly specific detection of hepatitis B virus genotypes B and C in clinical application. Front. Bioeng Biotechnol. 9. doi: 10.3389/fbioe.2021.743322 PMC8529042 34692662

[B4] China National Health Commission (2023). Guidelines for the diagnosis and treatment of Mycoplasma pneumoniae pneumonia in children (2023).) [EB/OL]. [2023-02-15]. Available at: Available at: http://www.nhc.gov.cn/yzygj/s7659/202302/8536e7db5cc7443eba13601e58d58861/files/b75c01f656c04653bfed27f0bb88b550.doc.

[B5] Daxboeck F. Krause R. Wenisch C. (2003). Laboratory diagnosis of Mycoplasma pneumoniae infection. Clin. Microbiol. Infect. 9 (4), 263–273. doi: 10.1046/j.1469-0691.2003.00590.x 12667235

[B6] Gong L. Jin Z. Liu E. Tang F. Yuan F. Liang J.. (2022). Highly sensitive and specific detection of mobilized colistin resistance gene mcr-1 by CRISPR-based platform. Microbiol. Spectrum 10 (5), e01884–e01822. doi: 10.1128/spectrum.01884-22 PMC9602551 36043860

[B7] Gootenberg J. S. Abudayyeh O. O. Lee J. W. Essletzbichler P. Dy A. J. Joung J.. (2017). Nucleic acid detection with CRISPR-Cas13a/C2c2. Science 356 (6336), 438–442. doi: 10.1126/science.aam9321 28408723 PMC5526198

[B8] Huang G. Huang Q. Xie L. Xiang G. Wang L. Xu H.. (2017). A rapid, low-cost, and microfluidic chip-based system for parallel identification of multiple pathogens related to clinical pneumonia. Sci. Rep. 7 (1), 6441. doi: 10.1038/s41598-017-06739-2 28743917 PMC5527024

[B9] Jacobs E. Ehrhardt I. Dumke R. (2015). New insights in the outbreak pattern of Mycoplasma pneumoniae. Int. J. Med. Microbiol. 305 (7), 705–708. doi: 10.1016/j.ijmm.2015.08.021 26319941

[B10] Jia N. Zhou J. Xiao F. Zheng B. Huang X. Sun C.. (2023). A CRISPR-Cas12a-Based platform for ultrasensitive, rapid, and highly specific detection of Mycoplasma pneumonia in clinical application. Front. Bioeng Biotechnol. 11. doi: 10.3389/fbioe.2023.1022066 PMC9887289 36733967

[B11] Jiang T. Wang Y. Jiao W. Song Y. Zhao Q. Wang T.. (2022). Recombinase polymerase amplification combined with real-time fluorescent probe for mycoplasma pneumoniae detection. J. Clin. Med. 11 (7), 1780. doi: 10.3390/jcm11071780 PMC9000086 35407388

[B12] Krafft C. Christy C. (2020). Mycoplasma pneumonia in children and adolescents. Pediatr. Rev. 41 (1), 12–19. doi: 10.1542/pir.2018-0016 31894069

[B13] Kutty P. K. Jain S. Taylor T. H. Bramley A. M. Diaz M. H. Ampofo K.. (2019). Mycoplasma pneumoniae among children hospitalized with community-acquired pneumonia. Clin. Infect. Dis. 68 (1), 5–12. doi: 10.1093/cid/ciy419 29788037 PMC6552676

[B14] Li S. Huang J. Ren L. Jiang W. Wang M. Zhuang L.. (2021). A one-step, one-pot CRISPR nucleic acid detection platform (CRISPR-top): Application for the diagnosis of COVID-19. Talanta 233, 122591. doi: 10.1016/j.talanta.2021.122591 34215080 PMC8197615

[B15] Li Y. Li S. Wang J. Liu G. (2019). CRISPR/Cas systems towards next-generation biosensing. Trends Biotechnol. 37 (7), 730–743. doi: 10.1016/j.tibtech.2018.12.005 30654914

[B16] Li L. Li S. Wu N. Wu J. Wang G. Zhao G.. (2019). HOLMESv2: A CRISPR-cas12b-assisted platform for nucleic acid detection and DNA methylation quantitation. ACS Synth Biol. 8 (10), 2228–2237. doi: 10.1021/acssynbio.9b00209 31532637

[B17] Li F. Xiao J. Yang H. Yao Y. Li J. Zheng H.. (2022). Development of a rapid and efficient RPA-CRISPR/cas12a assay for mycoplasma pneumoniae detection. Front. Microbiol. 13. doi: 10.3389/fmicb.2022.858806 PMC8965353 35369478

[B18] Liu G. Lin Q. Jin S. Gao C. (2022). The CRISPR-Cas toolbox and gene editing technologies. Mol. Cell. 82 (2), 333–347. doi: 10.1016/j.molcel.2021.12.002 34968414

[B19] Loens K. Goossens H. Ieven M. (2010). Acute respiratory infection due to Mycoplasma pneumoniae: current status of diagnostic methods. Eur. J. Clin. Microbiol. Infect. Dis 29 (9),1055–69. doi: 10.1007/s10096-010-0975-2 PMC7088226 20526788

[B20] Loens K. Ieven M. (2016). Mycoplasma pneumoniae: current knowledge on nucleic acid amplification techniques and serological diagnostics. Front. Microbiol. 7. doi: 10.3389/fmicb.2016.00448 PMC4814781 27064893

[B21] Mishra R. Cano E. Venkatram S. Diaz-Fuentes G. (2017). An interesting case of mycoplasma pneumonia associated multisystem involvement and diffuse alveolar hemorrhage. Respir. Med. Case Rep. 21, 78–81. doi: 10.1016/j.rmcr.2017.03.022 28413775 PMC5384885

[B22] Narita M. (2010). Pathogenesis of extrapulmonary manifestations of Mycoplasma pneumoniae infection with special reference to pneumonia. J. Infect. Chemother. 16 (3), 162–169. doi: 10.1007/s10156-010-0044-x 20186455

[B23] Notomi T. Okayama H. Masubuchi H. Yonekawa T. Watanabe K. Amino N.. (2000). Loop-mediated isothermal amplification of DNA. Nucleic Acids Res. 28 (12), E63. doi: 10.1093/nar/28.12.e63 10871386 PMC102748

[B24] Piepenburg O. Williams C. H. Stemple D. L. Armes N. A. (2006). DNA detection using recombination proteins. PloS Biol. 4 (7), e204. doi: 10.1371/journal.pbio.0040204 16756388 PMC1475771

[B25] Shankar E. M. Kumarasamy N. Balakrishnan P. Saravanan S. Solomon S. Vengatesan A.. (2007). Detection of pulmonary Mycoplasma pneumoniae infections in HIV-infected subjects using culture and serology. Int. J. Infect. Dis. 11 (3), 232–238. doi: 10.1016/j.ijid.2006.04.007 16914347

[B26] Smith C. B. Golden C. A. Kanner R. E. Renzetti A. D. Jr. (1980). Association of viral and Mycoplasma pneumoniae infections with acute respiratory illness in patients with chronic obstructive pulmonary diseases. Am. Rev. Respir. Dis. 121 (2), 225–232. doi: 10.1164/arrd.1980.121.2.225 6244766

[B27] Waites K. B. Xiao L. Liu Y. Balish M. F. Atkinson T. P. (2017). Mycoplasma pneumoniae from the respiratory tract and beyond. Clin. Microbiol. Rev. 30 (3), 747–809. doi: 10.1128/CMR.00114-16 28539503 PMC5475226

[B28] Wang Y. Wang Y. Ma A. J. Li D. X. Luo L. J. Liu D. X.. (2015). Rapid and sensitive isothermal detection of nucleic-acid sequence by multiple cross displacement amplification. Sci. Rep. 5, 11902. doi: 10.1038/srep11902 26154567 PMC4648395

[B29] Wang Y. Wang Y. Quan S. Jiao W. Li J. Sun L.. (2019). Establishment and application of a multiple cross displacement amplification coupled with nanoparticle-based lateral flow biosensor assay for detection of mycoplasma pneumoniae. Front. Cell Infect. Microbiol. 9. doi: 10.3389/fcimb.2019.00325 PMC6767991 31608243

[B30] Xiao F. Zhou J. Sun C. Huang X. Zheng B. Fu J.. (2022). Loop-mediated isothermal amplification coupled with nanoparticle-based biosensor: A rapid and sensitive method to detect mycoplasma pneumoniae. Front. Cell Infect. Microbiol. 12. doi: 10.3389/fcimb.2022.882855 PMC9299420 35873146

[B31] Zhou J. Xiao F. Fu J. Jia N. Huang X. Sun C.. (2023). Rapid detection of monkeypox virus by multiple cross displacement amplification combined with nanoparticle-based biosensor platform. J. Med. Virol. 95 (2), e28479. doi: 10.1002/jmv.28479 36609918

