# Supplementary material for: Rapid, ultrasensitive and highly specific diagnosis of Mycoplasma pneumoniae by a CRISPR-based detection platform
Source: Front Cell Infect Microbiol. 2023 Jul 27;13:1147142. doi: 10.3389/fcimb.2023.1147142 (PMC10414563; doi:10.3389/fcimb.2023.1147142)
Supplement: Supplementary file 1 [file DataSheet_1.docx]

**Title page**

**Rapid, Ultrasensitive and Highly Specific Diagnosis of *Mycoplasma Pneumoniae* by a CRISPR-Based Detection Platform**

Juan Zhou ^1^, Fei Xiao ^1^, Jin Fu ^1^, Nan Jia ^1^, Xiaolan Huang ^1^, Chunrong Sun ^1^, Zheng Xu ^1^, Yu Zhang ^1^, Dong Qu ^2*^ and Yi Wang ^1*^

^1^ Experimental research center, Capital Institute of pediatrics, Beijing, 100020, P.R. China.

^2^ Department of Critical Medicine, Children’s Hospital Affiliated Capital Institute of Pediatrics, Beijing 100020, P.R. China.

*Correspondence:

Prof. **Yi Wang**, [wildwolf0101@163.com](mailto:wildwolf0101@163.com) (Handing the Correspondence)

Prof. **Dong Qu**, qudong2012@126.com

**Tables**

**Table S1 Bacterial strains used in this study**

| **Pathogens** | **Source of strains ^a^** | **No. of strains** | **Results of MP-RPA-CRISPR assay ^b^** |
| --- | --- | --- | --- |
| *Mycoplasma pneumoniae* | ATCC | 1 | P |
| *Mycoplasma genitalium* | Isolated strains (CDC) | 1 | N |
| *Mycoplasma penetrans* | Isolated strains (CDC) | 1 | N |
| *Mycoplasma hominis* | Isolated strains (CDC) | 1 | N |
| *Mycoplasma primatum* | Isolated strains (CDC) | 1 | N |
| *Mycoplasma urealyticum* | Isolated strains (CDC) | 1 | N |
| *Neisseria meningitidis* | Isolated strains (CDC) | 1 | N |
| *Neisseria lactamica* | Isolated strains (CDC) | 1 | N |
| *Staphylococcus aureus* | Isolated strains (CDC) | 1 | N |
| *Streptococcus salivarius* | Isolated strains (CDC) | 1 | N |
| *Streptococcus pyogenes* | Isolated strains (CDC) | 1 | N |
| *Streptococcus pneumoniae* | Isolated strains (CDC) | 1 | N |
| *Streptococcus suis* | Isolated strains (CDC) | 1 | N |
| *Klebsiella pneumoniae* | Isolated strains (CDC) | 1 | N |
| *Stenotrophomonas maltophilia* | Isolated strains (CDC) | 1 | N |
| *Nocardia asteroides* | Isolated strains (CDC) | 1 | N |
| *Moraxella catarrhalis* | Isolated strains (CDC) | 1 | N |
| *Mycobacterium tuberculosis* | Isolated strains (CDC) | 1 | N |
| *Corynebacterium sriatum* | Isolated strains (CDC) | 1 | N |
| *Monilia albican* | Isolated strains (CDC) | 1 | N |
| *Bacillus cereus* | Isolated strains (CDC) | 1 | N |
| *Pseudomonas aeruginosa* | Isolated strains (CDC) | 1 | N |
| *Enterococcus faecium* | Isolated strains (CDC) | 1 | N |
| *Escherichia coli* | Isolated strains (CDC) | 1 | N |
| *Haemophilus influenzae* | Isolated strains (CDC) | 1 | N |
| *Bordetella pertussis* | Isolated strains (CDC) | 1 | N |

^a^CDC, Chinese center of disease control and prevention; ATCC, American type culture collection;

**Figures**


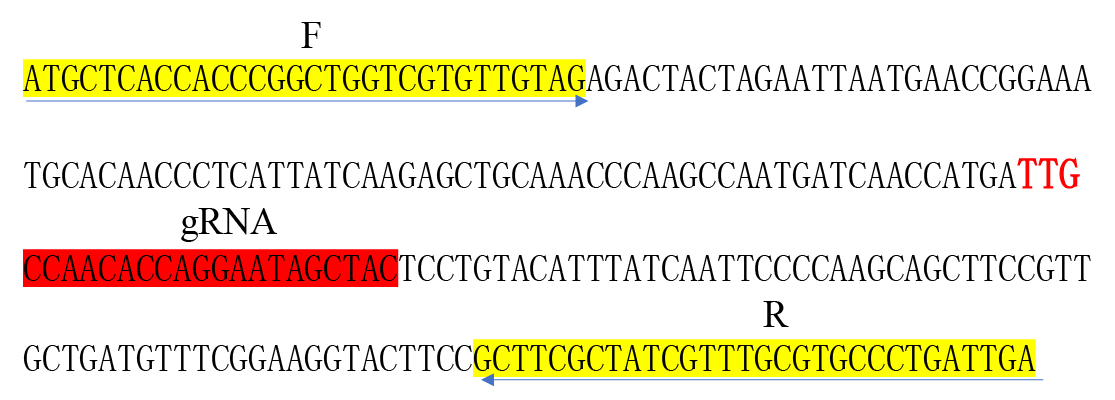


**Figure S1.** Primers and gRNA design of MP-RPA-CRISPR assay targeting the CARDS gene of MP strains. Right and left arrows show the sense and complementary sequences that are used.

**Figure S2. Confirmation of the cleavage activity of CRISPR-Cas12b effector. A**, Electrophoretic plot of RPA products before and after CRISPR-Cas12b-gRNA complex cleavage**.** Lines 1-2 referred to results before CRISPR-Cas12b-gRNA complex cleavage, lines 3-4 referred to results after CRISPR-Cas12b-gRNA complex cleavage; lines 1 and 3 referred to the positive control, and lines 2 and 4 referred to the blank control. M, 100 bp marker. **B**, Validation of accurate complex for non-target ssDNA cleavage. Changes in fluorescence signal in the presence or absence of CRISPR-Cas12b, gRNA or target DNA were recorded by real-time PCR platform to identify the accurate complex that works.

**
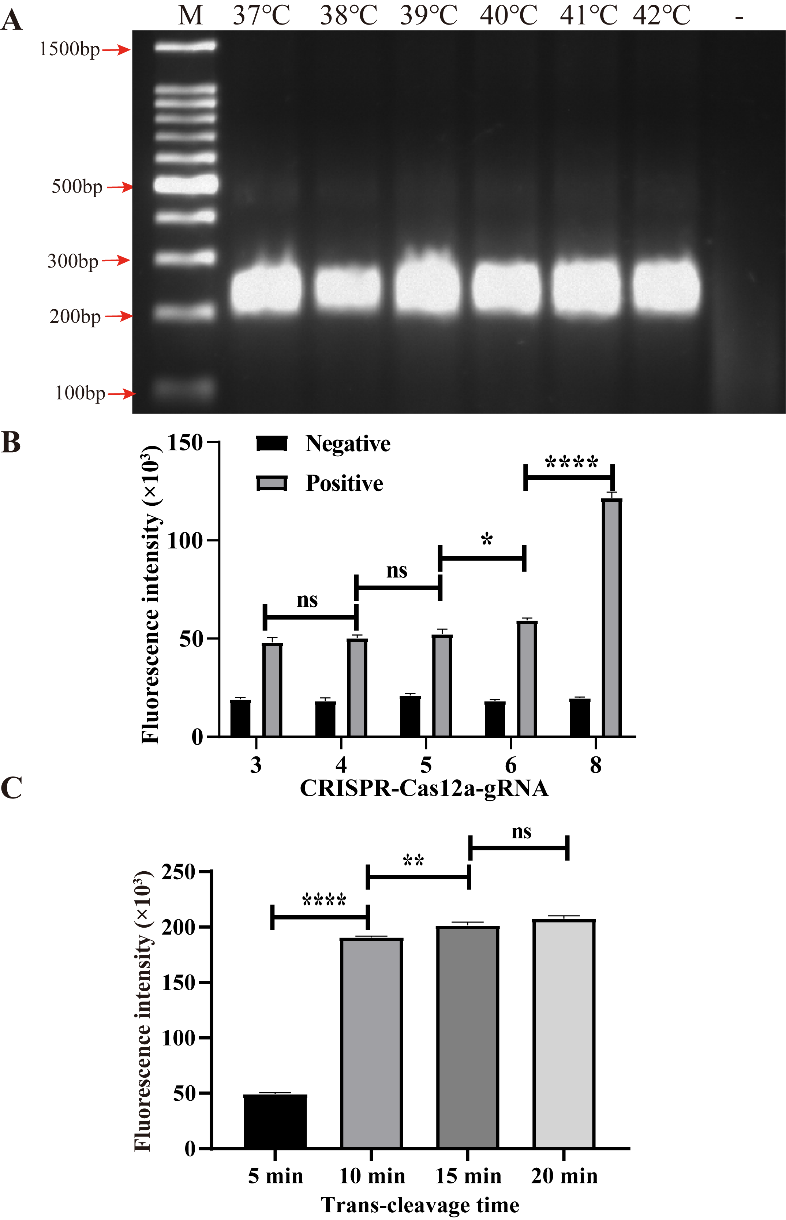
Figure S3. Optimization of the reaction conditions of MP-RPA-CRISPR assay. A,** Optimal reaction temperature for MP-RPA assay. The RPA products amplified at temperatures ranging from 37 to 42°C with 1 °C interval was detected by AGE method. 1-7 referred to the reaction temperature of 37, 38, 39, 40, 41, 42°C and the blank control DW. M, 100 bp marker. **B**, Optimal volume of CRISPR-Cas12b-gRNA complex for CRISPR-Cas12b trans-cleavage reaction. Results with a volume of 3, 4, 5, 6, 8 μl of CRISPR-Cas12b-gRNA complex were recorded and compared to obtain the optimal one for RPA products detection, respectively. **C**, Optimal reaction time for CRISPR-Cas12b trans-cleaving ssDNA. Results within 5 min, 10 min, 15 min and 20 min were compared and the optimal time was obtained. Each test has technical replicates, two-tailed Student's t test is used; bars represent mean ± SEM. Ns, no significance; ∗,p < 0.05; **, P< 0.01; ****, P <0.0001.

**
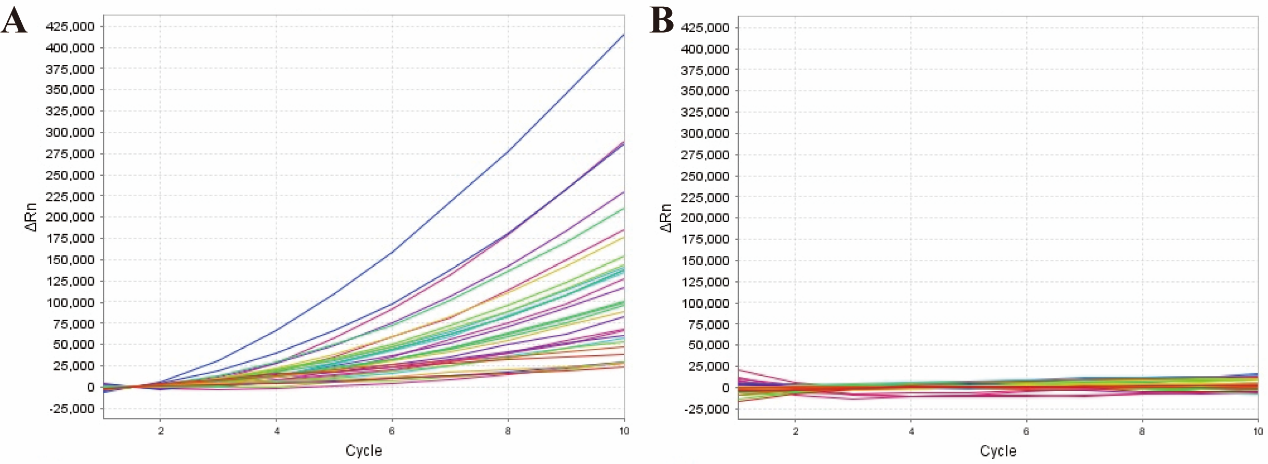
**

**Figure S4**. Clinical sample validation of the MP-RPA-CRISPR assay on the real-time PCR platform. **A**, Results of MP-positive clinical samples. **B**, Results of MP-negative clinical samples. The fluorescent signal density of positive samples were all higher than 25*10^3^, while the negative ones were all lower than this value.
